# Pharmacogenetic and clinical risk factors for bevacizumab-related gastrointestinal hemorrhage in prostate cancer patients treated on CALGB 90401 (Alliance)

**DOI:** 10.1038/s41397-024-00328-z

**Published:** 2024-03-04

**Authors:** Jai N. Patel, Chen Jiang, Kouros Owzar, Daniel L. Hertz, Janey Wang, Flora A. Mulkey, William K. Kelly, Susan Halabi, Yoichi Furukawa, Cameron Lassiter, Susan G. Dorsey, Paula N. Friedman, Eric J. Small, Michael A. Carducci, Michael J. Kelley, Yusuke Nakamura, Michiaki Kubo, Mark J. Ratain, Michael J. Morris, Howard L. McLeod

**Affiliations:** 1 Department of Cancer Pharmacology & Pharmacogenomics, Atrium Health Levine Cancer Institute, Charlotte, NC USA; 2 Alliance Statistics and Data Management Center, Duke University, Durham, NC USA; 3 Department of Biostatistics and Bioinformatics, Duke University, Durham, NC USA; 4 Department of Clinical Pharmacy, University of Michigan College of Pharmacy, Ann Arbor, MI USA; 5 Department of Biostatistics, Vanderbilt University Medical Center, Nashville, TN USA; 6 Sidney Kimmel Cancer Center, Thomas Jefferson University, Philadelphia, PA USA; 7 Institute of Medical Science, The University of Tokyo, Tokyo, Japan; 8 University of Maryland School of Nursing (Miltenyi Biotech at time of publication), Baltimore, MD USA; 9 Department of Pharmacology and Center for Pharmacogenomics, Northwestern University, Evanston, IL USA; 10 Helen Diller Family Comprehensive Cancer Center, University of California San Francisco, San Francisco, CA USA; 11 Johns Hopkins School of Medicine, Sidney Kimmel Comprehensive Cancer Center, Baltimore, MD USA; 12 Durham VA Medical Center/Duke University Medical Center, Durham, NC USA; 13 Center for Personalized Therapeutics, University of Chicago (Japanese Foundation for Cancer Research, Ariake, Tokyo at time of publication), Chicago, IL USA; 14 Riken Center for Integrative Medical Sciences (Haradoi Hospital, Fukuoka, Japan at time of publication), Kanagawa, Japan; 15 Division of Solid Tumor Oncology, Memorial Sloan Kettering Cancer Center, New York, NY USA; 16 Utah Tech University, St. George, UT USA

**Keywords:** Genetic association study, Risk factors

## Abstract

The objective of this study was to discover clinical and pharmacogenetic factors associated with bevacizumab-related gastrointestinal hemorrhage in Cancer and Leukemia Group B (Alliance) 90401. Patients with metastatic castration-resistant prostate cancer received docetaxel and prednisone ± bevacizumab. Patients were genotyped using Illumina HumanHap610-Quad and assessed using cause-specific risk for association between single nucleotide polymorphisms (SNPs) and gastrointestinal hemorrhage. In 1008 patients, grade 2 or higher gastrointestinal hemorrhage occurred in 9.5% and 3.8% of bevacizumab (*n* = 503) and placebo (*n* = 505) treated patients, respectively. Bevacizumab (*P* < 0.001) and age (*P* = 0.002) were associated with gastrointestinal hemorrhage. In 616 genetically estimated Europeans (*n* = 314 bevacizumab and *n* = 302 placebo treated patients), grade 2 or higher gastrointestinal hemorrhage occurred in 9.6% and 2.0% of patients, respectively. One SNP (rs1478947; HR 6.26; 95% CI 3.19–12.28; *P* = 9.40 × 10^−8^) surpassed Bonferroni-corrected significance. Grade 2 or higher gastrointestinal hemorrhage rate was 33.3% and 6.2% in bevacizumab-treated patients with the AA/AG and GG genotypes, versus 2.9% and 1.9% in the placebo arm, respectively. Prospective validation of these findings and functional analyses are needed to better understand the genetic contribution to treatment-related gastrointestinal hemorrhage.

## Introduction

Bevacizumab is a recombinant humanized monoclonal antibody targeting circulating vascular endothelial growth factor (VEGF) and is approved to treat many solid tumor malignancies [1–4]. VEGF is recognized as an essential regulator of normal and abnormal blood vessel growth [5]. It induces factor III, von Willebrand factor and tissue plasminogen activator, and is involved in the synthesis of nitric oxide and prostacyclin [6]. Inhibition of these processes may result in vascular instability contributing to thrombotic or hemorrhagic events, depending on the delicate balance of factors in a given patient [6, 7]. Furthermore, increased clot formation and vasoconstriction of the splanchnic vasculature can lead to bowel perforation [8].

The significance of bevacizumab-related hemorrhage is highlighted by a Food and Drug Administration (FDA) issued black box warning [9]. The estimated incidence of all-grade and high-grade (grade 3–5) hemorrhage is approximately 30 and 4%, respectively [10]. Although bevacizumab-related fatal adverse events are rare (2%), the most common cause of treatment-related fatality are hemorrhagic events (25%), primarily involving the lungs, central nervous system (CNS) and gastrointestinal system [11]. Studies of bevacizumab-treated patients also suggest an increased risk of hemorrhage at the site of the primary tumor [3, 12]. Furthermore, a recent pharmacovigilance study showed significantly higher risks of artery dissections or aneurysms in those receiving antiangiogenic drugs, including bevacizumab [13].

Clinical factors proposed to increase overall bleeding risk (independent of treatment) include recent major bleed, anemia, thrombocytopenia, cancer and antiplatelet/anticoagulant use [14]. Patients with cancer have increased bleeding risk when receiving anticoagulants compared to patients without cancer [15]. Genetic mutations can also result in hereditary bleeding disorders including hemophilia A (factor VIII deficiency), hemophilia B (factor IX deficiency), von Willebrand disease and other rare conditions [16]. One meta-analysis suggests an association between *MTHFR* gene polymorphisms and risk of intracranial hemorrhage [17], whereas other studies suggest a possible polygenic contribution to certain types of hemorrhagic stroke, such as subarachnoid hemorrhage [18], but the evidence base is lacking.

Given the lack of data on risk factors for bevacizumab-related hemorrhage, the primary objective of this study was to identify potential clinical and pharmacogenetic risk factors for bevacizumab-related grade 2 or higher (2+) gastrointestinal hemorrhage using a genome-wide approach in metastatic castration-resistant prostate cancer patients treated on Cancer and Leukemia Group B (CALGB) 90401 [19]. CALGB is now part of the Alliance for Clinical Trials in Oncology.

## Patients and methods

CALGB 90401 was a placebo-controlled double-blinded phase III trial that equally randomized men with metastatic castration-resistant prostate cancer to receive docetaxel 75 mg/m^2^ on day 1 of every 21-day cycle and prednisone 5 mg twice daily with or without bevacizumab 15 mg/kg on day 1 of every 21-day cycle, for up to two years [19]. Randomization was stratified by age, prior history of arterial events, and Halabi prognostic risk groups [20]. Patients were randomized to the trial from May 2005 to December 2007. Details on treatment and eligibility can be found in the original clinical trial publication [19]. Patients from the CALGB 90401 parent study who provided IRB-approved informed consent for the pharmacogenetic substudy (CALGB 60404) were eligible for the genome-wide association (GWAS) analysis.

Toxicity data were collected prospectively by the Alliance Statistics and Data Management Center at each treatment cycle on standardized forms that mandated reporting of all grade solicited toxicities, including gastrointestinal hemorrhage, and grade 3 or higher unsolicited toxicities, as defined by National Cancer Institute Common Toxicity Criteria for Adverse Events Version 3.0 (NCI-CTCAEv3.0) [21]. A gastrointestinal hemorrhage event for the analysis was defined as any grade 2+ gastrointestinal hemorrhage defined by the NCI-CTCAEv3.0 as “symptomatic and medical intervention or minor cauterization indicated” and considered possibly, probably, or definitely related to therapy per provider report. Medical history was collected from the patient on standardized prestudy forms that documented prior history of peptic ulcer disease (PUD), hemorrhage and/or gastrointestinal perforation, and smoking. Prior history of hemorrhage/gastrointestinal perforation was limited to events that occurred within five years prior to enrollment. Baseline hemoglobin and antiplatelet/anticoagulant use was recorded along with dose and frequency.

### Genotyping

A 10-mL sample of venous blood was collected prior to receiving treatment from all patients providing consent for the pharmacogenetic companion study [21]. Genotyping was conducted using the HumanHap610-Quad Genotyping BeadChip (Illumina Inc., CA, USA) at the RIKEN Center for Genomic Medicine (Yokohama City, Japan). Genotype data are available at dbGaP (https://www.ncbi.nlm.nih.gov/projects/gap/cgi-bin/study.cgi?study_id=phs001002.v1.p1). Patients with genotype call rates <95% and SNPs with call rates <99%, poor genotype clustering, indeterminate or unreliable loci, deviation from Hardy-Weinberg equilibrium (HWE) and non-autosomal loci were excluded prior to analysis. After removing SNPs with a HWE *p* < 10^−8^ and those with a minor allele frequency <0.05, 498,081 SNPs remained for the analysis. Eigensoft methods were used to visualize genetic ancestry and identify the subset of patients who were genetically estimated European [22], in whom the final pharmacogenetic analysis was conducted to avoid population stratification effects (i.e., when cases and controls are sampled from genetically different underlying populations where allele frequencies vary causing any associations observed to be due to sampling differences). Data quality was ensured by review of data by the Alliance Statistics and Data Management Center and by the study chairperson following Alliance policies.

### Statistical analysis

Statistical analyses were conducted by the Alliance Statistics and Data Management Center on a data set locked on January 28, 2014. All analyses were performed using R software (version 3.1.0) [23] and (version 2.37-7) [24], SAS software version 9.2 [25] and GenABEL (version 1.8-0) [26]. A competing risks model was utilized where the event of interest (grade 2+ gastrointestinal hemorrhage) was subject to three dependent informative censoring mechanisms: progression/death, other treatment-terminating adverse events, or ‘other’, which included lost to follow-up, withdrawal for reasons other than toxicity, or incomplete information [21]. Patients who did not complete two years of therapy due to any of these competing risks, prior to experiencing the event of interest, were informatively censored at the cumulative bevacizumab cycles received. All analyses were conducted by considering the influence of bevacizumab therapy and cause-specific hazard.

A multivariable Cox regression model [27] was used to identify baseline covariates associated with gastrointestinal hemorrhage, including age (continuous), history of PUD (yes vs. no), history of hemorrhage (yes vs. no), history of smoking (yes vs. no), hemoglobin (continuous) and baseline antiplatelet/anticoagulant use (yes vs. no).

The Log-rank statistic was used to test the association of all 498,081 SNPs that passed quality control with cumulative bevacizumab/placebo cycle at occurrence of grade 2+ gastrointestinal hemorrhage. A dominant genetic model was assumed as data suggest it is more robust against sampling deviations from HWE frequencies than the additive model and the higher likelihood within a GWAS to identify variants with low minor allele frequency (MAF) [28]. The Bonferroni-corrected p-value threshold for significance was set at 1 × 10^−7^. The 1000 SNPs that had the strongest association with gastrointestinal hemorrhage were adjusted for baseline covariates with *p* < 0.1 in multivariable analysis. The 100 SNPs with the strongest association after adjustment for clinical covariates were further interrogated for biological function and cross referenced with previously described SNPs associated with hereditary bleeding disorders. HaploReg v2 [29] was used to investigate linkage disequilibrium (LD) between SNPs and altered transcription factors (high LD defined as r^2^ ≥ 0.90).

PrediXcan is a gene-based association method that correlates imputed gene expression with the phenotype of interest to identify genes involved in the etiology of the phenotype [30]. This methodology was used to test the genetically predicted gene expression using Depression Genes and Networks (DGN) whole blood elastic net model for association with grade 2+ gastrointestinal hemorrhage. The whole blood model was used because of its relevance to hemorrhage and large number of genotyped samples. The Bonferroni-corrected p-value threshold for significance was set at 9 × 10^−5^, based on 556 genes tested. To search for cumulative genetic effects and provide insight into mechanisms, functions, and pathways involved with bevacizumab-related hemorrhage, genes with an unadjusted *p* < 0.05 from the PrediXcan results were included in a pathway enrichment analysis using Ingenuity Pathway Analysis version 17199142 (Ingenuity ® Systems, Inc, www.ingenuity.com).

A phenome-wide association study (PheWAS) was performed to assess the association between genetic polymorphisms with phenotypes by linking genotyping from BioVu, an independent de-identified DNA data bank at Vanderbilt University, to a broad range of electronic medical record–derived clinical phenotypes [31]. Briefly, phecodes are defined by hierarchical groupings of ICD-9 and ICD-10 codes. Cases are defined as individuals with 2 or more phecodes on unique dates and controls are individuals who do not have a phecode of interest. We used phecodes version 1.2 (available at https://phewascatalog.org/phecodes). Genotyping was performed using the Illumina MEGA array and quality control protocol developed by the Vanderbilt Epidemiology Center in cooperation with Vanderbilt Technologies for Advanced Genomics Analysis and Research Design. Further details on the PheWAS approach can be found in prior publications [32]. The PheWAS was conducted on 72,083 individuals of European ancestry and performed using a logistic regression, controlling for age and sex. Phecode associations with SNPs yielding uncorrected *P-*values lower than 0.05 were summarized. The Bonferroni-corrected *p*-value threshold for significance was set at 3.0 × 10^−5^, based on 1508 Phecodes tested.

## Results

**Fig. 1:**
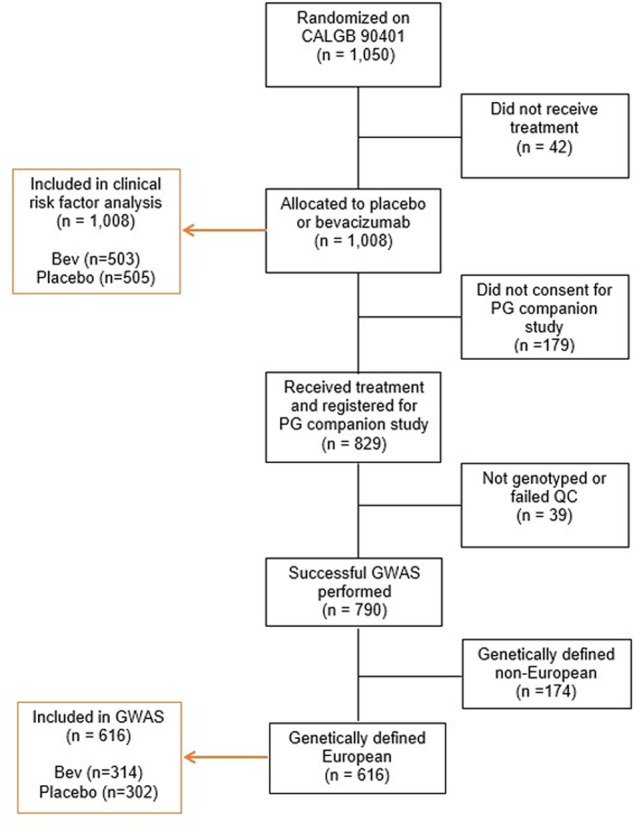
Consort diagram. Of the 1050 patients randomized on CALGB (Alliance) 90401, 42 did not receive treatment resulting in 1008 patients who were included in the clinical risk factor analysis (503 received bevacizumab; 505 received placebo). Of these, 179 did not consent for the pharmacogenetic companion study and 39 were not genotyped and/or failed quality control, resulting in 790 patients with successful genotyping performed. Of these, 616 genetically-estimated Europeans who received treatment were included in the GWAS (314 received bevacizumab; 302 received placebo).

CALGB 90401 enrolled 1050 patients, of whom 1008 received treatment and were included in the clinical analysis (503 received bevacizumab and 505 received placebo). Patients in both arms received a median of eight cycles The GWAS included 616 genetically estimated European patients (314 received bevacizumab and 302 received placebo) (Fig. 1). Baseline demographics were similar between the clinical and GWAS cohorts, and bevacizumab and placebo treated patients (Table 1). Using a time-to-event competing risk model, the rate of grade 2+ gastrointestinal hemorrhage by treatment arm (bevacizumab and placebo) in the clinical cohort was 9.5% (48/503) and 3.8% (19/505) (HR = 2.7; 95% CI 1.53–4.93; *P* < 0.001) and in the GWAS cohort was 9.6% (30/314) and 2.0% (6/302) (HR = 5.2; 95% CI 2.09–15.52; *P* < 0.001), respectively.

**Table 1:** Patient baseline demographics.

Baseline demographics		Clinical cohort		GWAS cohort	
		Bevacizumab (*N* = 503)	Placebo (*N* = 505)	Bevacizumab (*N* = 314)	Placebo (*N* = 302)
Age (years)	Median (range)	68.7 (41.7–92.9)	69.2 (41.7–93.5)	69.0 (42.1–92.9)	68.9 (41.7–93.5)
Self-reported race	White	447 (88.9%)	440 (87.1%)	314 (100%)	302 (100%)
	African American	48 (9.5%)	58 (11.5%)	N/A	N/A
	Other	8 (1.6%)	7 (1.4%)	N/A	N/A
ECOG performance status	0	284 (56.4%)	281 (55.6%)	170 (54.1%)	171 (56.6%)
	1	199 (39.6%)	203 (40.2%)	134 (42.7%)	123 (40.7%)
	2	20 (4.0%)	21 (4.2%)	10 (3.2%)	8 (2.7%
Cumulative bevacizumab/ placebo cycles	Median (range)	8 (0–39)	8 (0–40)	8 (0–39)	8 (1–40)
History of peptic ulcer disease	Yes	26 (5.2%)	21 (4.2%)	14 (4.5%)	13 (4.3%)
	No	451 (89.7%)	458 (90.7%)	282 (89.8%)	277 (91.7%)
History of hemorrhage^a^	Yes	17 (3.4%)	20 (4.0%)	11 (3.5%)	12 (4.0%)
	No	462 (91.8%)	464 (91.9%)	287 (91.4%)	281 (93.1%)
Antiplatelet/anticoagulant use	Yes	293 (58.3%)	307 (60.8%)	191 (60.8%)	190 (62.9%)
	No	210 (41.7%)	195 (38.6%)	123 (39.2%)	111 (36.8%)
Hemoglobin	≤10 mg/dL	32 (6.4%)	34 (6.7%)	17 (5.4%)	15 (5.0%)
	>10 mg/dL	471 (93.6%)	471 (93.3%)	297 (94.6%)	287 (95.0%)
History of smoking	Yes	223 (44.3%)	225 (44.6%)	139 (44.3%)	130 (43.1%)
	No	253 (50.3%)	255 (50.5%)	157 (50.0%)	160 (53.0%)

Note percentages do not always add up to 100% due to missing data.

*ECOG* Eastern Cooperative Oncology Group.

^a^Prior history of hemorrhage was limited to events that occurred within 5 years prior to enrollment and included gastrointestinal/genitourinary hemorrhage and/or gastrointestinal perforation.

### Clinical risk factors

In multivariable analysis (Table 2), bevacizumab treatment was confirmed to increase grade 2+ gastrointestinal hemorrhage risk compared to placebo in the clinical (HR 2.88; 95% CI: 1.64–5.06; *P* < 0.001) and GWAS cohorts (HR = 5.70; 95% CI: 2.17–14.97; *P* < 0.001). Of the remaining covariates, increasing age as a continuous variable also increased grade 2+ gastrointestinal hemorrhage risk in the clinical (HR 1.05; 95% CI 1.02–1.09; *P* = 0.002) and GWAS cohorts (HR = 1.06; 95% CI: 1.01–1.12; *P* = 0.01). History of PUD was significantly associated with grade 2+ gastrointestinal hemorrhage in the univariate analysis but did not maintain significance in the multivariable analysis (clinical cohort: HR 2.20, 95% CI: 0.98–4.90, *P* = 0.055; GWAS cohort: HR 2.61, 95% CI 0.87–7.83, *P* = 0.09). There was lack of association between history of hemorrhage, antiplatelet/anticoagulant use, smoking, and hemoglobin with increased hemorrhage risk (all *p* > 0.05).

**Table 2:** Univariate and multivariable analysis of grade 2+ gastrointestinal hemorrhage by treatment and clinical risk factors.

Risk factor	Cause-specific hazard ratio (95% CI) *P*-value			
	Clinical cohort (*N* = 1008)		GWAS cohort (*N* = 616)	
	Univariate	Multivariable	Univariate	Multivariable
Bevacizumab treatment	2.50 (1.47–4.26) <0.001	2.88 (1.64–5.06) <0.001	4.67 (1.94–11.21) <0.001	5.70 (2.17–14.97) <0.001
Age^a^	1.05 (1.02–1.08) 0.001	1.05 (1.02–1.09) (0.002)	1.05 (1.01–1.10) 0.01	1.06 (1.01–1.12) 0.01
History of peptic ulcer disease	2.44 (1.11–5.36) 0.03	2.20 (0.98–4.90) 0.055	2.97 (1.04–8.46) 0.04	2.61 (0.87–7.83) 0.09
History of hemorrhage^b^	1.16 (0.36–3.71) 0.80	0.97 (0.30–3.14) 0.96	1.60 (0.38–6.70) 0.52	0.98 (0.22–4.33) 0.98
Antiplatelet/ anticoagulant use	1.34 (0.81–2.21) 0.26	1.29 (0.76–2.19) 0.35	1.25 (0.63–2.51) 0.52	1.14 (0.54–2.42) 0.72
History of smoking	1.27 (0.77–2.09) 0.34	1.31 (0.79–2.17) 0.29	1.22 (0.61–2.44) 0.57	1.32 (0.65–2.66) 0.45
Hemoglobin^a^	0.94 (0.82–1.09) 0.45	0.93 (0.79–1.08) 0.34	0.92 (0.75–1.13) 0.45	0.94 (0.75–1.17) 0.57

^a^Age and hemoglobin as continuous variables.

^b^Prior history of hemorrhage includes gastrointestinal/genitourinary hemorrhage and/or gastrointestinal perforation.

### SNP analysis

**Fig. 2:**
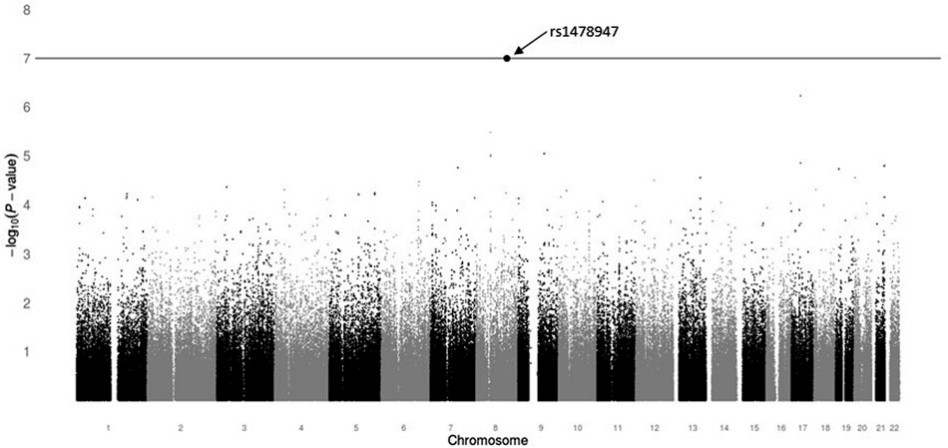
Manhattan plot of *p*-values for the association of all SNPs that passed quality control with occurrence of grade 2+ hemorrhage. Of 498,081 SNPs tested, one intergenic SNP (rs1478947, MAF = 0.06; HR 6.26; 95% CI 3.20–12.28; *P* = 9.40 × 10^−8^) surpassed Bonferroni-corrected significance (*P* = 1.0 × 10^−7^; solid line) for association with grade 2+ gastrointestinal hemorrhage.

**Fig. 3:**
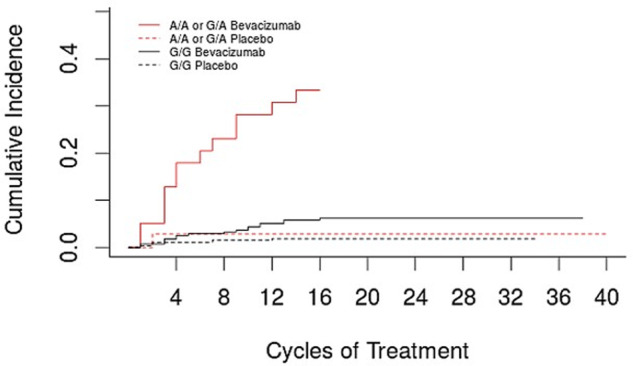
Rate of grade 2+ gastrointestinal hemorrhage stratified by rs1478947 genotype and treatment arm. The rate of gastrointestinal hemorrhage (from time of treatment initiation to treatment end, up to 2 years) was 33.3% (13/39) and 6.2% (17/275) in bevacizumab-treated patients with the AA/AG and GG genotypes, respectively, while the incidence in the placebo arm was 2.9% (1/35) and 1.9% (5/267). Because of the low MAF, only one patient carried the AA genotype. This patient was randomized to receive bevacizumab and experienced a grade 3 gastrointestinal hemorrhage during cycle 3, which resulted in removal from study treatment.

The top 10 SNPs ranked by *p*-value of their association with grade 2+ gastrointestinal hemorrhage, adjusted for bevacizumab treatment, age, and history of PUD, are reported in Table 3, along with the rsID, gene annotation, MAF, and adjusted cause-specific HR. Of 498,081 SNPs tested, one intergenic SNP (rs1478947, MAF = 0.06; HR 6.26; 95% CI 3.19–12.28; *P* = 9.40 × 10^−8^) surpassed Bonferroni-corrected significance (*P* = 1.0 × 10^−7^) for association with grade 2+ gastrointestinal hemorrhage (see Fig. 2 for Manhattan plot). The rate of grade 2+ gastrointestinal hemorrhage was 33.3% (13/39) and 6.2% (17/275) in bevacizumab-treated patients with the AA/AG and GG genotypes, respectively, while the incidence in the placebo arm was 2.9% (1/35) and 1.9% (5/267) (Fig. 3). The one patient who carried the AA genotype was randomized to receive bevacizumab and experienced a grade 3 gastrointestinal hemorrhage during cycle 3, which resulted in treatment withdrawal. There was no significant interaction between SNP and treatment arm (*P* = 0.23).

**Table 3:** Top 10 SNPs by adjusted *p*-value.

Rank	rsID	Chromosome	Gene	Allele	MAF	Adjusted HR (95% CI)	Adjusted *P*-value
1	rs1478947	8	NA	G > A	0.06	6.26 (3.19–12.28)	9.40 × 10^−8^
2	rs8064614	17	AATF	G > A	0.20	3.60 (2.18–5.95)	5.83 × 10^−7^
3	rs10513906	18	NA	G > A	0.26	3.67 (2.17–6.21)	1.23 × 10^−6^
4	rs12546435^a^	8	SNTG1	G > A	0.14	3.19 (1.96–5.19)	3.26 × 10^−6^
5	rs10993698	9	SYK	C > A	0.13	3.61 (2.05–6.35)	8.83 × 10^−6^
6	rs203898^a^	8	SNTG1	C > A	0.13	3.03 (1.86–4.95)	9.36 × 10^−6^
7	rs203927^a^	8	SNTG1	G > A	0.13	3.03 (1.86–4.95)	9.36 × 10^−6^
8	rs203959^a^	8	SNTG1	G > A	0.14	3.03 (1.85–4.95)	9.84 × 10^−6^
9	rs203946^a^	8	SNTG1	G > A	0.14	3.02 (1.85–4.94)	1.01 × 10^−5^
10	rs11870749	17	AATF	A > G	0.22	3.04 (1.84–5.01)	1.40 × 10^−5^

*SNP* single nucleotide polymorphism, *HR* cause-specific hazard ratio, *MAF* minor allele frequency, *NA* not applicable, meaning the SNP was not found within a gene (i.e., intergenic).

^a^rs12546435, rs203898, rs203927, rs203959, rs203946 are all in high linkage disequilibrium (LD) (r^2^ ≥ 0.90), none of which are in LD with the top SNP rs1478947.

### Predicted gene expression analysis

The top 10 genes ranked according to the statistical association between genotype-predicted gene expression (i.e., PrediXcan) and grade 2+ gastrointestinal hemorrhage by adjusted *p*-value, along with the corresponding adjusted cause-specific HR, are reported in Supplementary Table S1. No genes were significantly associated with gastrointestinal hemorrhage risk after adjusting for multiple comparisons. The top ranked gene, *ITPA*, encodes the protein inosine triphosphate pyrophosphohydrolase (*ITPA*, HR = 5.77; 95% CI 2.29–14.56; *P* = 2.03  × 10^−4^).

Supplementary Table S2 summarizes the top canonical pathways enriched from the gene-based pathway analysis, their respective *p*-values, and genes mapped to each pathway. Of these, Heme Biosynthesis from Uroporphyrinogen-III I, the third most highly enriched pathway (*P* = 0.0038), was identified as having possible biological relevance to hemorrhage.

### PheWAS

Of 1508 diagnosis phecodes tested, 66 are summarized with a *p* < 0.05, none of which were directly related to gastrointestinal hemorrhage, but several possibly indirectly related and associated with anemia and thrombocytopenia (Supplementary Table S3). No associations surpassed Bonferroni correction.

## Discussion

To our knowledge, this report is the first to investigate both clinical and pharmacogenetic risk factors for bevacizumab-related gastrointestinal hemorrhage, which was collected prospectively from a large, randomized phase III trial. Consistent with previous reports, [12, 15, 33] bevacizumab treatment significantly increased gastrointestinal hemorrhage risk compared to placebo. Increasing age, prior PUD, and an intergenic SNP (rs1478947) were associated with cause-specific gastrointestinal hemorrhage risk in CALGB (Alliance) 90401.

Incidence rates of grade 2 (and thus grade 2 or higher) gastrointestinal hemorrhage are infrequently reported in prior bevacizumab studies given these trials typically reported grade 3 or higher events and combine grade 1 and 2; however, grade 2 events were included in this analysis given the high risk for severe complications, hospitalization, and the need for medical intervention. While bevacizumab is not approved or used in patients with prostate cancer, a previous study reported that 1.7% and 3.4% of advanced ovarian cancer patients receiving chemotherapy plus placebo or chemotherapy plus bevacizumab experienced a grade 2+ gastrointestinal adverse event, including perforation, fistula, hemorrhage or necrosis, respectively; however, these patients were relatively younger and fewer had a history of PUD [34]. Two large meta-analyses of bevacizumab-treated patients have reported incidence rates of overall grade 3+ hemorrhage of 2.8% and 3.5% [10, 35], one of which reported the highest incidence for gastrointestinal hemorrhage (2.3%) [10]. The incidence of grade 3+ gastrointestinal hemorrhage in the CALGB 90401 parent study (~6.0%) was relatively high compared to previous meta-analyses – this may be partially due to administration of higher bevacizumab doses and an older population. Compared to other solid tumors, patients with prostate cancer have similar rates of severe bleeding (<5% and mostly hematuria) [36, 37], like the ~4% observed in our clinical cohort receiving placebo. However, bleeding rates are generally increased in cancer patients receiving anticoagulant treatment, those with metastatic disease, and patients with gastrointestinal primary malignancies and/or liver metastases [38].

Older patients and those with a history of PUD were at increased risk for gastrointestinal hemorrhage during treatment. Consistent with these findings, previous studies have identified increasing age and history of PUD as risk factors for gastrointestinal hemorrhage in patients receiving other drugs known to increase bleeding risk, such as aspirin and nonsteroidal anti-inflammatory drugs [39]. Previous studies have also demonstrated that bevacizumab treatment does not increase the risk of severe bleeding in patients receiving therapeutic anticoagulation compared to those not receiving anticoagulation [40]. We did not observe an association between anticoagulant/antiplatelet use and gastrointestinal hemorrhage risk. Thrombocytopenia was not investigated as a risk factor since patients were required to have a minimum platelet count of 100,000/µl for enrollment and only 5% of patients had a platelet count of ≤150,000/µl.

A genome-wide analysis was undertaken to identify SNPs associated with gastrointestinal hemorrhage risk. Previously reported SNPs associated with hereditary bleeding disorders, such as hemophilia A and B and von Willebrand’s disease [41], were not observed among the top 1000 SNPs in this analysis. No genes related to the top 100 SNPs were found to have a direct biological relationship to hemorrhage risk; however, one intergenic SNP (rs1478947) surpassed genome-wide significance. Rs1478947 is located on chromosome 8 at position 97471007 (Genome Reference Consortium Human Build 38)with a MAF 0.05 in HapMap CEU samples and 0.06 in the study population. Although rs1478947 genotype significantly increased gastrointestinal hemorrhage risk regardless of treatment arm, there was more than a 5-fold increase in toxicity rate in patients with the AA/AG versus GG genotype receiving bevacizumab compared to an approximately 1.5-fold increase in patients receiving placebo (Fig. 3). This suggests the effect of rs1478947 may be potentiated in patients receiving bevacizumab; however, this could not be confirmed directly via statistical interaction analysis.

Although the exact mechanism by which rs1478947 might increase gastrointestinal hemorrhage risk remains unclear, rs1478947 is in complete LD (r^2^ = 1.0) with rs1478948 (located 30 base pairs upstream from rs1478947), variations of which may alter the binding motif for transcription factor hepatocyte nuclear factor-4 (HNF4).^26^ HNF4A (located on chromosome 20) is localized to intestine, kidneys, and liver, whereas HNF4G is located on chromosome 8 (same as rs1478947) and primarily expressed in the small bowel [42]. Prior reports demonstrated that HNF4 exerts a positive regulatory effect on clotting factor VII (fVII) expression and binding of this transcription factor is critical for normal fVII function [43, 44]. Mutations in the HNF4 binding site within the fVII promoter result in altered binding, severe fVII deficiency and an increased risk of bleeding [44–46]. Further, mutations in the HNF4 binding site of factor IX also cause severe bleeding disorders [47]. The Leyden phenotype of the severe bleeding disorder hemophilia B is caused by several point mutations within the promoter region, of which a number map in the HNF4 binding site [47]. It could be hypothesized that increased gastrointestinal damage by presence of rs1478947 combined with bevacizumab may increase bleeding risk in the gastrointestinal tract.

PrediXcan was used to test genetically predicted gene expression for association with gastrointestinal hemorrhage [30]. The top ranked gene, *ITPA*, did not surpass Bonferroni corrected significance. Polymorphisms in *ITPA* have been shown to increase the risk of drug-induced anemia and thrombocytopenia [48, 49], which are general risk factors for increased bleeding. Ingenuity Pathway Analysis of genes from the PrediXcan results identified several enriched canonical pathways, including Heme Biosynthesis from Uroporphyrinogen III I (Supplementary Table S2). Uroporphyrinogen III is the precursor for synthesis of vitamin B12, chlorophyll, and heme. Defects in this pathway can result in porphyrias, which primarily result in neurological symptoms [50]; however, abnormalities in heme synthesis may also result in increased risk of hemorrhage as well as oxidative stress post-hemorrhage [51].

PheWAS uses a reverse genetics approach to associate genetic variants with phenotypes by linking a database of deidentified genotypes to a broad range of electronic medical record-derived clinical phenotypes. The most common categories were endocrine/metabolic, hematopoietic, mental disorders, and circulatory system, but none surpassed statistical significance (Supplementary Table S3).

This analysis has limitations, including limited sample size, lack of available independent cohorts of bevacizumab-treated patients with prospectively collected toxicity data and banked DNA for replication, and absence of functional studies to determine biological plausibility. The success of any pharmacogenomics GWAS depends on effect size, allele frequency of genetic variants that impact the event of interest, the population (including race/ethnicity), and study design. GWAS by nature have little power due to multiple testing, thus requiring large sample sizes (e.g., >1000) to detect significant associations with low MAF. Importantly, large disease susceptibility GWAS (with sample sizes much larger than this analysis) have discovered and validated genetic predictors of complex phenotypes, including obesity [52], diabetes [53], and others, in intergenic regions of the DNA that have not yet been linked to a biological mechanism. It remains unknown how these variations mechanistically influence outcome, although one may speculate transcriptional regulation, long-range promoter regulation, variations in micro- or non-coding RNA, and other factors. Databases such as Encyclopedia of DNA Elements (Encode; https://genome.ucsc.edu/ENCODE), The Genotype-Tissue Expression (GTEx) project (http://www.gtexportal.org/home), MiTranscriptome (http://mitranscriptome.org), and others, are built to increase the understanding of the biological consequences of intergenic variants; however, currently available data and understanding are still limited. Functional studies are critical to determine the biological plausibility of GWAS findings. Induced pluripotent stem cell-derived endothelial cell (iPSC-EC) responses may be a novel approach to evaluate sensitivity to anti-VEGF agents [54]. Future studies can use iPSC-ECs to assess gene expression profiles associated with drug sensitivity or specific phenotypes. Lastly, this study was limited to genetically estimated Europeans based on the population enrolled on the parent prospective clinical trial and there is a critical need to conduct GWAS in diverse populations. Notably, the frequency of rs1478947 in Africans is 0.09 based on 1000 Genomes, thus replication in this population is critical.

In conclusion, this analysis confirms the increased risk of gastrointestinal hemorrhage associated with bevacizumab treatment and increasing age. A potentially novel association was noted between an intergenic SNP, rs1478947, with gastrointestinal hemorrhage; however, the relatively low MAF, lack of a direct biological relationship to hemorrhage risk based on current literature, lack of functional studies, and unavailable replication cohort limit the interpretation of rs1478947 as a potential predictor of hemorrhage risk. This intronic variant, while removed during transcription, may serve a regulatory function, or involved in alternative splicing. It is in complete LD with rs1478948, which we hypothesize may have putative functionality in altering the binding motify of HNF4 (previously reported to be associated with hemorrhage risk). This finding may be particularly important in other solid tumors that are more prone to gastrointestinal hemorrhage and in which bevacizumab is more commonly used, like certain gastrointestinal malignancies (e.g., colorectal cancer). Future studies should focus on identifying and replicating factors that influence treatment-related hemorrhage risk, with the goal of building an algorithm to prospectively predict toxicity risk prior to initiating treatment. Understanding both clinical and pharmacogenetic risk factors for treatment-related hemorrhage is essential to mitigate risks and reduce the burden of this prevalent, and potentially fatal, complication.

### Supplementary information

Supplementary Material

## Data Availability

Genotype data are available at dbGap (http://www.ncbi.nlm.nih.gov/projects/gap/cgi-bin/study.cgi?study_id¼phs001002.v1.p1). Details regarding the CALGB 90401 parent study can be found at: Kelly WK, Halabi S, Carducci M, et al. Randomized, double-blind, placebo-controlled phase III trial comparing docetaxel and prednisone with or without bevacizumab in men with metastatic castration-resistant prostate cancer: CALGB 90401. J Clin Oncol. 2012;30: 1534-1540. Additional data may be requested from the corresponding author (JNP).
