# Supplementary material for: Pharmacogenetic and clinical risk factors for bevacizumab-related gastrointestinal hemorrhage in prostate cancer patients treated on CALGB 90401 (Alliance)
Source: Pharmacogenomics J. 2024 Mar 4;24(2):6. doi: 10.1038/s41397-024-00328-z (PMC10912014; doi:10.1038/s41397-024-00328-z)
Supplement: Supplementary file 1 — Supplementary Material [file 41397_2024_328_MOESM1_ESM.docx]

**Supplemental Material**

**Supplemental Table 1** The top 10 genes ranked according to the statistical association between genotype-predicted gene expression (i.e., PrediXcan) and grade 2+ gastrointestinal hemorrhage by adjusted p-value

| Rank | Gene | Adjusted HR | Adjusted p-value^a^ |
| --- | --- | --- | --- |
| 1 | ITPA | 5.77 | 2.03 x 10^-4^ |
| 2 | MATN2 | 10.75 | 2.18 x 10^-4^ |
| 3 | DTX1 | 1.04 x 10^3^ | 3.10 x 10^-4^ |
| 4 | SPNS3 | 8.85 x 10^-6^ | 3.60 x 10^-4^ |
| 5 | SMARCA5 | 2.05 x 10^7^ | 4.51 x 10^-4^ |
| 6 | CA3 | 2.44 x 10^-3^ | 5.43 x 10^-4^ |
| 7 | ALDH18A1 | 70.88 | 5.50 x 10^-4^ |
| 8 | APRT | 2.14 x 10^-3^ | 6.60 x 10^-4^ |
| 9 | C2orf84 | 0.011 | 8.36 x 10^-4^ |
| 10 | AARSD1 | 1.54 x 10^-3^ | 9.00 x 10^-4^ |

HR - cause-specific hazard ratio

**Supplemental Table 2** Top 10 Canonical Pathways Enriched in Ingenuity Pathway Analysis from Gene-based GWAS (PrediXcan)

| Ingenuity Canonical Pathways | p-value | Genes |
| --- | --- | --- |
| Stearate Biosynthesis I (Animals) | 0.0003 | ACOT2, ACOT4, SLC27A3, ACOT7, ACSBG2, SLC27A4 |
| Acyl-CoA Hydrolysis | 0.003 | ACOT2, ACOT4, ACOT7 |
| Heme Biosynthesis from Uroporphyrinogen-III I | 0.0038 | PPOX, CPOX |
| Proline Biosynthesis I | 0.0038 | PYCRL, ALDH18A1 |
| Fatty Acid Activation | 0.0039 | SLC27A3, ACSBG2, SLC27A4 |
| D-myo-inositol-5-phosphate Metabolism | 0.0043 | PLCD1, CA3, PLCE1, PDCD1, NUDT3, NUDT12, PPFIBP2, CILP, PPP1R14A, UBLCP1 |
| Superpathway of Inositol Phosphate Compounds | 0.0045 | PLCD1, CA3, PIP5K1A, PLCE1, PDCD1, NUDT3, NUDT12, PPFIBP2, CILP, PPP1R14A, PI4K2B, UBLCP1 |
| D-myo-inositol (1,4,5)-Trisphosphate Biosynthesis | 4.57E-03 | PLCD1, PIP5K1A, PLCE1, PI4K2B |
| Trans, trans-farnesyl Diphosphate Biosynthesis | 0.0062 | IDI1, IDI2 |
| Dendritic Cell Maturation | 0.0063 | PLCD1, COL1A1, PLCE1, CD80, LEPR, NFKBIE, IL15, CD83, IL6, TNFRSF1B, FCGR3A, FCGR3B |

**Supplemental Table 3. Top diagnoses using PheWAS for rs1478947**

| **ICD code** | **Diagnosis** | **Cases** | **Controls** | **Odds ratio** | **P-value** | **Category** |
| --- | --- | --- | --- | --- | --- | --- |
| 132 | Infestation (lice, mites) | 91 | 65535 | 2.237 | 0.002 | infectious diseases |
| 281.12 | Other vitamin B12 deficiency anemia | 656 | 47208 | 1.439 | 0.002 | hematopoietic |
| 279.7 | Other immunological findings | 239 | 65535 | 1.658 | 0.004 | endocrine/metabolic |
| 743.12 | Senile osteoporosis | 976 | 59500 | 0.6784 | 0.005 | musculoskeletal |
| 290.16 | Vascular dementia | 173 | 57508 | 0.2406 | 0.005 | mental disorders |
| 290.1 | Dementias | 1350 | 57508 | 0.7419 | 0.007 | mental disorders |
| 728.7 | Fasciitis | 941 | 54910 | 1.304 | 0.009 | musculoskeletal |
| 681.2 | Cellulitis and abscess of face/neck | 450 | 59772 | 1.432 | 0.009 | dermatologic |
| 411.1 | Unstable angina (intermediate coronary syndrome) | 2084 | 53183 | 1.208 | 0.012 | circulatory system |
| 264 | Lack of normal physiological development | 2021 | 51432 | 0.7918 | 0.012 | endocrine/metabolic |
| 907 | Injuries to the nervous system | 344 | 65535 | 1.479 | 0.012 | injuries & poisonings |
| 240 | Simple and unspecified goiter | 420 | 57359 | 0.5855 | 0.013 | endocrine/metabolic |
| 772.6 | Facial weakness | 176 | 62786 | 1.646 | 0.016 | symptoms |
| 429.9 | Cardiac complications, not elsewhere classified | 352 | 57086 | 0.5645 | 0.017 | circulatory system |
| 198.6 | Secondary malignancy of bone | 1709 | 55345 | 1.206 | 0.017 | neoplasms |
| 522 | Diseases of pulp and periapical tissues | 160 | 65535 | 0.3432 | 0.018 | digestive |
| 522.5 | Periapical abscess | 144 | 65535 | 0.3046 | 0.018 | digestive |
| 647.1 | Infections of genitourinary tract during pregnancy | 168 | 39426 | 1.646 | 0.019 | pregnancy complications |
| 836 | Traumatic arthropathy | 162 | 65535 | 1.657 | 0.020 | injuries & poisonings |
| 429 | Ill-defined descriptions and complications of heart disease | 2478 | 57086 | 0.8383 | 0.021 | circulatory system |
| 798.1 | Chronic fatigue syndrome | 590 | 43050 | 1.332 | 0.022 | symptoms |
| 514.2 | Solitary pulmonary nodule | 1412 | 61768 | 1.213 | 0.024 | respiratory |
| 930 | Allergic reaction to food | 716 | 57098 | 0.7116 | 0.024 | injuries & poisonings |
| 755.1 | Congenital deformities of feet | 164 | 65535 | 1.632 | 0.024 | congenital anomalies |
| 644 | Anemia during pregnancy | 120 | 39891 | 1.731 | 0.025 | pregnancy complications |
| 292.6 | Hallucinations | 180 | 57508 | 0.4266 | 0.026 | mental disorders |
| 290.3 | Other persistent mental disorders due to conditions classified elsewhere | 737 | 57508 | 0.72 | 0.027 | mental disorders |
| 284.1 | Pancytopenia | 1310 | 47208 | 1.216 | 0.028 | hematopoietic |
| 272.12 | Hyperglyceridemia | 505 | 45949 | 1.347 | 0.028 | endocrine/metabolic |
| 379.9 | Pain, swelling or discharge of eye | 225 | 62424 | 0.4956 | 0.028 | sense organs |
| 430 | Intracranial hemorrhage | 1172 | 60319 | 0.7804 | 0.029 | circulatory system |
| 687 | Symptoms affecting skin | 2113 | 54877 | 1.169 | 0.030 | dermatologic |
| 288.11 | Neutropenia | 1881 | 53631 | 1.177 | 0.030 | hematopoietic |
| 290.12 | Dementia with cerebral degenerations | 110 | 57508 | 0.2834 | 0.031 | mental disorders |
| 752 | Nervous system congenital anomalies | 556 | 65535 | 0.685 | 0.031 | congenital anomalies |
| 523.32 | Chronic periodontitis | 115 | 65535 | 1.722 | 0.031 | digestive |
| 613 | Other nonmalignant breast conditions | 1889 | 65535 | 0.8271 | 0.032 | genitourinary |
| 295.1 | Schizophrenia | 287 | 39546 | 1.447 | 0.033 | mental disorders |
| 281 | Other deficiency anemia | 1193 | 47208 | 1.222 | 0.033 | hematopoietic |
| 961.1 | Poisoning/allergy of sulfonamides | 833 | 54612 | 0.7455 | 0.033 | injuries & poisonings |
| 509.2 | Respiratory insufficiency | 1941 | 49819 | 0.8313 | 0.034 | respiratory |
| 394.1 | Mitral valve stenosis and aortic valve stenosis | 506 | 58431 | 0.6791 | 0.035 | circulatory system |
| 353.1 | Nerve plexus lesions | 126 | 62312 | 0.3506 | 0.038 | neurological |
| 284 | Aplastic anemia | 1502 | 47208 | 1.19 | 0.038 | hematopoietic |
| 281.1 | Megaloblastic anemia | 871 | 47208 | 1.251 | 0.039 | hematopoietic |
| 726.3 | Bursitis | 736 | 54910 | 0.7376 | 0.039 | musculoskeletal |
| 270.38 | Other specified disorders of plasma protein metabolism | 215 | 65535 | 0.5176 | 0.040 | endocrine/metabolic |
| 575.2 | Obstruction of bile duct | 500 | 65398 | 0.6863 | 0.040 | digestive |
| 442.11 | Abdominal aortic aneurysm | 734 | 59099 | 0.7412 | 0.040 | circulatory system |
| 110.2 | Dermatomycoses | 109 | 62131 | 0.3033 | 0.040 | infectious diseases |
| 369.2 | Eye infection, viral | 185 | 62343 | 0.48 | 0.040 | sense organs |
| 618.6 | Vaginal enterocele, congenital or acquired | 195 | 38011 | 1.531 | 0.041 | genitourinary |
| 331.9 | Cerebral degeneration, unspecified | 265 | 54934 | 0.574 | 0.041 | neurological |
| 117.4 | Aspergillosis | 119 | 62131 | 1.672 | 0.041 | infectious diseases |
| 277.5 | Other disorders of lipoid metabolism | 172 | 65535 | 1.552 | 0.042 | endocrine/metabolic |
| 155.1 | Malignant neoplasm of liver, primary | 503 | 65535 | 1.319 | 0.042 | neoplasms |
| 728.71 | Contracture of palmar fascia [Dupuytren's disease] | 151 | 54910 | 0.4381 | 0.046 | musculoskeletal |
| 618.5 | Prolapse of vaginal vault after hysterectomy | 285 | 38011 | 1.43 | 0.046 | genitourinary |
| 807 | Fracture of ribs | 782 | 59475 | 0.7565 | 0.047 | injuries & poisonings |
| 574.12 | Cholelithiasis with other cholecystitis | 265 | 65398 | 0.5837 | 0.047 | digestive |
| 604.3 | Peyronie's disease | 115 | 26486 | 0.3677 | 0.047 | genitourinary |
| 647 | Infectious and parasitic complications affecting pregnancy | 181 | 39426 | 1.522 | 0.047 | pregnancy complications |
| 713 | Arthropathy associated with other disorders classified elsewhere | 238 | 62812 | 1.453 | 0.048 | musculoskeletal |
| 365.2 | Primary angle-closure glaucoma | 214 | 64760 | 1.484 | 0.048 | sense organs |
| 274.11 | Gouty arthropathy | 805 | 65535 | 0.7614 | 0.049 | endocrine/metabolic |
| 317 | Alcohol-related disorders | 1813 | 51291 | 0.8387 | 0.049 | mental disorders |
